# Supplementary material for: Epidermolysis-Bullosa-Associated Squamous Cell Carcinomas Support an Immunosuppressive Tumor Microenvironment: Prospects for Immunotherapy
Source: Cancers (Basel). 2024 Jan 22;16(2):471. doi: 10.3390/cancers16020471 (PMC10814073; doi:10.3390/cancers16020471)
Supplement: Supplementary file 1 [file cancers-16-00471-s001.zip › cancers-2749768-supplementary.pdf]

Supplementary Materials

Table S1. Disease-causing mutations and clinical characteristics of EB patients

Patient	Gender	Mutated gene	Mutations cDNA	Mutations protein	Clinical description of SCCs	Therapy	Course and outcome of SCC	Alive age at last visit / Deceased at age (years
DEB-P1	F	*COL7A1*	c.4888C>T;5743_5744dupCG	p.Arg1630*;Gly1916Valfs*90	More than 80 SCCs mainly on the limbs	Immunotherapy Pembrolizumab Cetuximab	Highly aggressive SCC	Deceased age 51
DEB-P2	F	*COL7A1*	c.425A>G; 425A>G	p.?;?	More than 20 SCC on the limbs	Excision	Multiple and recurrent	Alive age 22
DEB-P3	F	*COL7A1*	c.497dup;2005C>T	p.Val168Glyfs*12; Arg669*				Deceased age 49
DEB-P5	F	*COL7A1*	c.4027C>T;7023G>A	p.Arg1343*;?				Alive age 56
DEB-P6	M	*COL7A1*	c.1637-240_3255del4064	p.?;?	Recurrent SCC on both hands	Excision	Metastasis	Deceased age 22
DEB-P7	M	*COL7A1*	c.425A>G;425A>G	p.?;?	Recurrent SCC on both hands, legs	Excision	Squamous cell carcinoma of the esophagus (ref)	Deceased age 42
DEB-P8	F	*COL7A1*	c.3551-3T>G;7987G>T	p.?;Glu2663*				Deceased age 26
DEB-P9	M	*COL7A1*	c.425A>G;1837C>T	p.?;Arg613*	Recurrent SCC on the right hand, SCC of the foot	Excision Cemiplimab, Cetuximab	Lymph node metastasis, new cutaneous SCC (ref)	Deceased age 28
DEB-P10	M	*COL7A1*	c.425A>G;425A>G	p.?;?	Recurrent SCC on the right hand, SCC of the sole, back	Excision Cemiplimab	Squamous cell carcinoma of the esophagus Cutaneous SCC stable under Cemiplimab/ref)	Deceased age 42
KEB-P1	M	*FERMT1*	c.456dupA;456dupA	p.Asp153Argfs*4;Asp153Argfs*4	Lip and oral mucosa	Excision, anti-EGFR (Cetuximab)	Multiple recurrences, metastasis	Deceased age 35

KEB-P2	M	*FERMT1*	c.958-1G>A;958-1G>A	p.?;?	Lip	Excision	No recurrence	Alive age 51
KEB-P3	M	*FERMT1*	c.958-1G>A;958-1G>A	p.?;?	Lip and hand	Excisions	Multiple recurrences of the SCC on the hand, metastasis	Deceased age 64
KEB-P4	M	*FERMT1*	c.910G>T; 910G>T	p.Glu304*;Glu304*	Foot (ankle) and leg	Excision, anti-EGFR (Cetuximab), anti-PD-1 (Nivolumab, Pembrolizumab)	Multiple recurrences, metastasis	Deceased age 50
KEB-P5	F	*FERMT1*	g.80929_89169del	p.?;?	Lip and hand	Excisions	Multiple recurrences of the SCC on the lip	Deceased age 60
KEB-P6	M	*FERMT1*	c.1209C>G;1209C>G	p.Tyr403*;Tyr403*	Hand	Excision	No recurrence	Alive age 61
KEB-P7	F	*FERMT1*	c.328C>T;328C>T	p.Arg110*;Arg110*	Hand	Excision	No recurrence	NA

Legend: c., cDNA; F, female; fs, frame shift; g., genomic DNA; M, male; ?, consequence not known; NA, not available

Table S2. Underlying diseases of the immunosuppressed patients in this study

**Organ transplantation**	**34**	**80.49**
Kidney	22	53.66
Liver	1	2.44
Lung	2	4.88
Heart	1	2.44
SCT[1]	8	17.07
**Inflammatory / autoimmune disease**	**4**	**9.76**
Rheumatoid arthritis	2	4.88
Psoriasis	1	2.44
Bullous pemphigoid	1	2.44
**Congenital immune deficiency**	**1**	**2.44**
$CD4^+$ T cell defect	1	2.44
**Solid tumour**	**1**	**2.44**
Squamous cell carcinoma of the nasopharynx	1	2.44
**Haematological malignancy**	**1**	**2.44**
B-CLL[2]	1	2.44

[1]Stem cell transplant; [2]Chronic lymphocytic leukemia.

Table S3. Immunosuppressive drugs used in the IS[1]-SCC patients in this study

Type of immunosuppression	Number	Percentage
**mTOR-Inhibitors**	**5**	**12.2**
Everolimus	3	7.32
Sirolimus	2	4.88
**Calcineurin inhibitors**	**24**	**58.54**
Tacrolimus	17	41.46
Cyclosporine	7	17.07
**Purine synthesis inhibitors**	**25**	**60.98**
Mycophenolic acid	20	48.78
Azathioprine	5	12.2
**Antimetabolites**	**3**	**7.32**
Methotrexate	3	7.32
**Alkylating agents**	**1**	**2.44**
Chlorambucil	1	2.44
**Alkylating-like agent**	**1**	**2.44**
Cisplatin	1	2.44
**Proteasome inhibitor**	**1**	**2.44**
Bortezomib	1	2.44
**NA[2]**	**3**	**7.32**
**Combination therapy**	**23**	**56.1**
Cyclosporine + Mycophenolic acid	4	9.76
Tacrolimus + Mycophenolic acid	14	34.15
Tacrolimus + Everolimus + Mycophenolic acid	1	2.44
Tacrolimus + Azathioprine	1	2.44
Sirolimus + Mycophenolic acid	2	4.88
Everolimus + Tacrolimus	1	2.44

[1]immunosupressed; [2]not available.

Table S4. Immunohistochemical staining reagents and methods

Primary antibody	Source	Antigen retrieval	Primary antibody incubation	Detection
IDO	Cell Signaling Technology	TRS[1], pH 9.0 (Dako)	60 min, RT	Biotin-conjugated secondary antibody (Dako REAL™ Link, Biotinylated Secondary Antibodies) visualized with a alkaline phosphatase/RED chromogen (Dako REAL™ Detection System, Rabbit/Mouse (Code K5005))
PD-1	R&D	TRS, pH 6.0 (Dako)	60 min, RT	Biotin-conjugated secondary rabbit anti-goat antibody (vector Labs) visualized with a alkaline phosphatase/RED chromogen (Dako REAL™ Detection System, Rabbit/Mouse (Code K5005)
PD-L1	Cell Signaling Technology	TRS, pH 9.0 (Dako)	60 min, RT	Biotin-conjugated secondary antibody (Dako REAL™ Link, Biotinylated Secondary Antibodies) visualized with a alkaline phosphatase/RED chromogen (Dako REAL™ Detection System, Rabbit/Mouse (Code K5005)
CD4	Invitrogen	TRS, pH 9.0 (Dako)	60 min, RT	Biotin-conjugated secondary antibody (Dako REAL™ Link, Biotinylated Secondary Antibodies) visualized with a alkaline phosphatase/RED chromogen (Dako REAL™ Detection System, Rabbit/Mouse (Code K5005)
CD8	Biolegend	TRS, pH 9.0 (Dako)	60 min, RT	Biotin-conjugated secondary antibody (Dako REAL™ Link, Biotinylated Secondary Antibodies) visualized with a alkaline phosphatase/RED chromogen (Dako REAL™ Detection System, Rabbit/Mouse (Code K5005)
CD68	Biolegend	TRS pH 9.0 (Dako)	60 min, RT	Biotin-conjugated secondary antibody (Dako REAL™ Link, Biotinylated Secondary Antibodies) visualized with a alkaline phosphatase/RED chromogen (Dako REAL™ Detection System, Rabbit/Mouse (Code K5005)
LAG-3	Novus Biologicals	TRS, pH 9.0 (Dako)	60 min, RT	Biotin-conjugated secondary antibody (Dako REAL™ Link, Biotinylated Secondary Antibodies) visualized with a alkaline phosphatase/RED chromogen (Dako REAL™ Detection System, Rabbit/Mouse (Code K5005)
TIM-3	Cell Signaling Technology	TRS, pH 9.0 (Dako)	60 min, RT	Biotin-conjugated secondary antibody (Dako REAL™ Link, Biotinylated Secondary Antibodies) visualized with a alkaline phosphatase/RED chromogen (Dako REAL™ Detection System, Rabbit/Mouse (Code K5005)

[1]target retrieval solution.

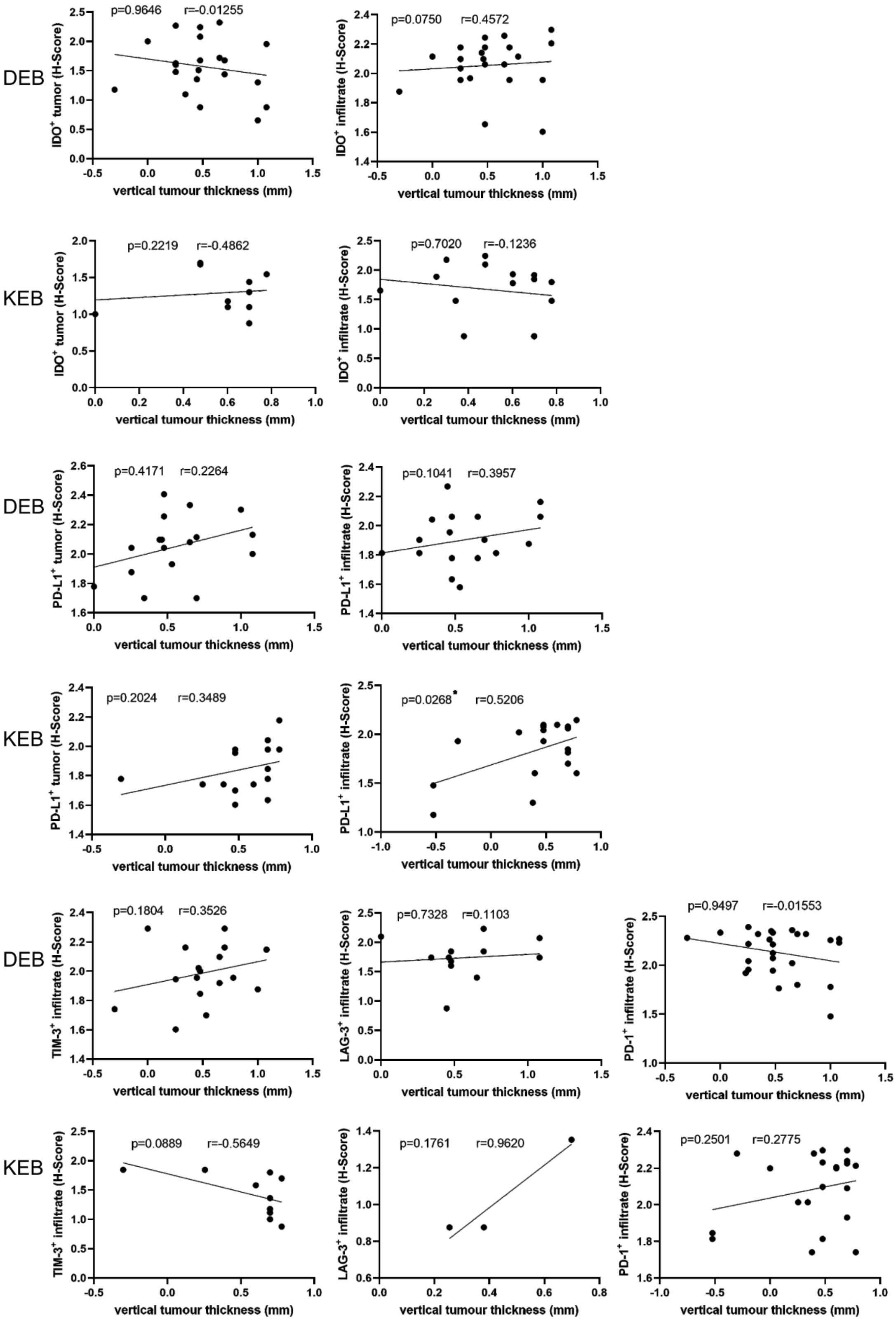

Figure S1: Correlation of immune marker expression with vertical tumor thickness. Statistical analysis shows how the expression of the markers (y-axis) in tumor cells and stromal cells is affected by vertical tumor thickness (x-axis) using a simple linear regression model. DEB=dystrophic epidermolysis bullosa, KEB=Kindler epidermolysis bullosa, Statistical significance was noted as *p<0.05, **p<0.01, ***p<0.001. r=pearson correlation coefficient. Measured values of X and Y-axis were logarithmized before statistical analysis.
